# Health as Complete Well-Being: The WHO Definition and Beyond

**DOI:** 10.1093/phe/phad017

**Published:** 2023-07-27

**Authors:** Thomas Schramme

**Affiliations:** Department of Philosophy, University of Liverpool, Gillian Howie House, Mulberry Street, Liverpool, L69 7SH, UK

## Abstract

The paper defends the World Health Organisation (WHO) definition of health against widespread criticism. The common objections are due to a possible misinterpretation of the word *complete* in the descriptor of health as ‘complete physical, mental and social well-being’. *Complete* here does not necessarily refer to perfect well-being but can alternatively mean exhaustive well-being, that is, containing all its constitutive features. In line with the alternative reading, I argue that the WHO definition puts forward a holistic account, not a notion of perfect health. I use historical and analytical evidence to defend this interpretation. In the second part of the paper, I further investigate the two different notions of health (holistic health and perfect health). I argue that both ideas are relevant but that the holistic interpretation is more adept for political aims.

## Introduction

‘Health is a state of complete physical, mental and social well-being and not merely the absence of disease or infirmity’ (World Health Organisation [WHO], 1948: 100). In this paper, I argue that this famous WHO definition of health is fully adequate. Criticism that has been levied against it is based on a specific interpretation that is not the only alternative. In addition to defending the WHO definition, I will discuss two different meanings of the concept of health, which can lead to confusion if not properly kept apart. This is important, for historical and analytical reasons, because the WHO definition can indeed be interpreted in different ways and because we need to get to grips with the differences between types of definitions of health. My second aim in this paper is hence to explain and to properly keep apart two different conceptualisations of health.^1^

As regards the WHO definition, I will claim that critics have read the word *complete* in the phrase ‘complete physical, mental and social well-being’ in a way that goes against the likely intentions of the draftees of the definition. The common objections, for instance, accusing the WHO definition of utopianism and overreach, are based on an implicit assumption, according to which *complete* is a quantitative term. In other words, critics assume that the phrase means that health is a state of well-being to the largest degree. I will call this interpretation *perfect health*. So, the critics claim that the WHO identifies health with the largest degree of well-being, that is, with perfect well-being or—in less technical terms—with happiness.

However, the term *complete* can also have a qualitative meaning.^2^ When we say that something is a complete specimen of its kind, then we mean that it has all the features that are constitutive of it. For instance, a complete dinner is one that contains a starter, a main dish and a dessert. Accordingly, complete well-being might be understood as a state that is exhaustive of all constitutive features of well-being. These are, according to the WHO, physical, mental and social aspects. I will call this *holistic health*.^3^ In brief, I will claim that the WHO endorses a holistic account of health, not a perfectionist account.^4^

In the second section, I briefly introduce the most important objections to the WHO definition. They have mainly to do with an alleged confusion of health with happiness, which then purportedly leads to a form of medicalisation of human life. In the third section, I discuss the likely intentions behind the WHO definition. I do this by referring to the two readings mentioned before, perfect health and holistic health. There are systematic and historical reasons as to why the WHO plausibly intended a holistic interpretation of health. In the fourth section, I discuss the two interpretations of health in their own right. I introduce their purposes and some objections to either notion. As is the case with many concepts we use, there is no single right or wrong conceptualisation of health. However, I argue that a holistic concept of health is better suited for the purposes of the WHO and more generally for political and economic agendas.

## Criticism of the WHO Definition

The health definition of the WHO has often been dismissed by philosophers of medicine and medical scientists (for an overview, see Leonardi, 2018). One of the main reasons has been the alleged confusion of health and happiness, that is, a state of complete well-being.^5^ If health is understood as happiness, it has been argued, there are many highly problematic consequences, most importantly the medicalisation of people’s lives. After all, health is also interpreted as a basic human right in the same document: ‘The enjoyment of the highest attainable standard of health is one of the fundamental rights of every human being without distinction of race, religion, political belief, economic or social condition’ (WHO, 1948: 100). If people fall short of the ideal of perfection, that is, if they are not in a state of complete well-being, their health ought to be enhanced. With health care being an important instrument to reach health, the lives of people seem to fall under the remit of health-related institutions, especially medicine, in all their aspects. For instance, if someone is sad, they lack health in the sense of complete well-being. Accordingly, following the WHO constitution, they apparently have a justified claim to be made healthy, that is, happy, potentially by using mood-enhancing drugs or other medical means.

A prominent and influential critique of the WHO definition stems from Daniel Callahan: ‘[T]he most specific complaint about the WHO definition is that its very generality, and particularly its association of health and general well-being as a positive ideal, has given rise to a variety of evils. Among them are the cultural tendency to define all social problems, from war to crime in the streets, as “health” problems’ (Callahan, 1973: 78; see also Kass, 1975: 14, for a very similar critique). This is an example of the critique of overreach (cf. Bickenbach, 2017: 962), that is, of applying a medical concept to areas that pose other types of problems than healthcare problems.

Another problem that has repeatedly been pointed out is the utopianism of the definition. It seems that ‘[t]he requirement for complete health “would leave most of us unhealthy most of the time”’ (Huber *et al*., 2011: 235; quoting Smith, 2008; see also Saracci, 1997: 1409, 1409; Card, 2017). This can specifically be deemed problematic in relation to people with disabilities, chronic diseases and people of advanced age. They would by definition permanently be missing out on health and accordingly on well-being. However, such a view seems to conflict with the perspectives of relevant groups of people themselves (Fallon and Karlawish, 2019: 1104).

Despite the widespread criticism from many different disciplinary backgrounds, the WHO never amended their definition of health. It seems that they did not see a need to change their point of view. In the following section, I will argue that the critique is indeed based on a misunderstanding of the WHO’s perspective.

## Interpreting the WHO Definition of Health

As explained, I will argue that the WHO defines health as holistic health, not as perfect health. To bolster this claim about the intentions of the institution, I need to consider the history of its constitution. In this section, I will therefore rely on historical documents, which are in the public domain. In addition, I have benefitted from an enormously helpful recent publication by Lars Thorup Larsen (2022), who gives a detailed account specifically of the genealogy of the WHO definition, based on archival research.

An important fact that supports my reading of the WHO’s intentions is that the word *complete* was only inserted into the definition at the very final stages of its conception. It is fairly obvious that it was as a form of editorial amendment, not a substantial change, because otherwise it would have required extensive debate. If the word *complete* would have fixed the intended definition of health to a perfectionist account, this would have either stirred up a debate or would have had to be uncontroversial. However, there is no evidence in the relevant documents that the draftees of the WHO constitution definitely understood health as perfection. The term *complete*, according to my reading, was rather intended to clarify the phrase ‘physical, mental and social well-being’, the latter of which had been part of the definition since the drafting period.^6^ The word *complete* summarises and jointly describes the three aspects of well-being. It also adds a rhetorical contrast to the second part of the sentence that denies the sufficiency of the absence of disease or infirmity for health. A perhaps better way to express the notion would have been to state that: health is a state of complete well-being, that is, a state that comprises physical, mental and social elements. But this locution would not have worked straightforwardly in a one-sentence definition, which was apparently aimed at by the WHO.

The late arrival of the term *complete* of course does not present conclusive evidence that the WHO did not intend to push an account of perfect health. The historical records are not sufficient in this respect. The final draft of the constitution, which had been penned by the Technical Preparatory Committee, was discussed at a meeting in New York City in 1946.^7^ The relevant draft definition reads: ‘Health is not only the absence of disease, but also a state of physical and mental well-being and fitness resulting from positive factors, such as adequate feeding, housing and training’ (WHO, 1947: 58). The final version, which was eventually adopted, had been prepared by the so-called Committee I, which ‘had given careful consideration to amendments submitted by the delegations of South Africa, Mexico, Australia, Belgium, Netherlands, Chile, United Kingdom, Iran, China, Philippines, Poland, Venezuela, United States of America and Canada’ (WHO, 1948: 44). Unfortunately, there are no published minutes or other forms of evidence in relation to this decisive period—decisive, as far as the introduction of the term *complete* is concerned. We simply do not know who added the word. This would have been important, though, to get a better grasp of the intentions behind the addition.^8^

Importantly, many members of the Technical Preparatory Committee, who had been involved to different degrees in the drafting of the WHO constitution, came from a public health background (Farley, 2008: 12ff.; Cueto *et al*., 2019: 39ff.). Renowned proponents of so-called social medicine, such as Andrija Štampar, René Sand, Karl Evang and Thomas Parran, were leading members of the drafting group. This is significant because public health usually has a different understanding of the concept of health than clinical medicine. Whereas for the latter, health can be defined as absence of disease (Smith, 2008), that is, in absolute terms, *health* in public health is a multifarious and scalar notion (Schramme, 2017; Valles, 2018: 31ff.).

In clinical medicine, health is often understood as absence of disease. This makes sense because the focus is on individual patients. These either have a disease or not. Patients might suffer from a more or less severe disease, but that does not mean that they are more or less diseased than others. Similarly, health over and above the absence of disease is not usually the focus of clinical medicine. If there is no disease, then that is sufficient to establish health. There is no need to refer to health in a positive way, that is, to define it in its own terms.

In contrast, public health scientists usually refer to populations. In their parlance, chosen populations can be more or less healthy than comparison groups. For instance, it might be declared that mine workers are less healthy than millionaires. This does not mean that all mine workers acutely suffer from a disease; rather, it means that they are more likely to fall ill, due to their circumstances of life. Public health has traditionally studied the causes of disease and has made big strides in the prevention of disease. Accordingly, its focus is upstream, as it is sometimes put (Marmot, 2010: 41; Venkatapuram, 2011: 189), towards the conditions that make disease more likely. Health becomes a dispositional term that allows for different grades.

From a public health perspective, it is fairly obvious that health is ‘more than the absence of disease’. It is more in the sense of additionally requiring dispositional elements, not because it is a quantitatively better condition than medical normality (i.e. the absence of disease). People who live in destitute circumstances might not suffer from a disease, but they are often lacking in terms of a sufficient disposition to maintain minimal health.

The public health perspective, therefore, is a gradual perspective on health, allowing parlance of more and less health, or being healthier than others. Although such a perspective does not necessarily lead to an account of perfect health, it is nevertheless compatible with the latter. People with a perfect health disposition—marked by a very low probability to fall ill—might accordingly be deemed in a state of perfect health. Importantly, falling below the ideal point of perfection on a scale does not imply having a disease. In other words, not being perfectly healthy would not constitute a condition of being *un*healthy; it would merely mean being *less* healthy than others (Schramme 2019: 29ff.). This shows that some of the criticism levied against the WHO definition, even if understood as a perfectionist account, is implausible. More specifically, it does not necessarily follow that, for instance, people with disabilities would be constantly deemed unhealthy because they lack perfect health. As explained, health is not a binary term according to the relevant perspective.

So far, I have argued that the WHO definition is supposed to allow for grades of health. For that purpose, it takes its cue from public health perspectives, though I do not want to claim that it is identical to it. After all, the WHO definition still incorporates the traditional medical perspective on health as absence of disease. There are, nevertheless, important qualms to do with the notion of perfect health. The WHO refers to health as a state of well-being and this might itself be deemed problematic. To be sure, the conceptual connection between health and the good life for human beings has long been established (Temkin, 1973).^9^ The connection also makes sense from an experiential point of view. Health has indeed to do with how we fare. Still, if we read the definition as a perfectionist account of health, it would define health as perfect well-being. If that were the case, this would apparently lead to the alleged dangerous confusion of health and happiness mentioned earlier. After all, sufficient health but not happiness seems to be the business of welfare state institutions. It is true, of course, that health care from a public health perspective includes vastly more than just medical care, especially aspects to do with work, education and the environment. Yet, we normally see good reasons to restrict the remit of state institutions to a form of needs provision, basic security and enablement of self-determination (cf. Goodin, 1988: 363ff.). So, if perfect health were the focus of the state, it would probably end up becoming unjustifiably expansive.

I do not believe that the WHO is guilty of this charge. To be sure, there are reasons for thinking that a public health perspective occasionally tends towards an expansive view of health politics (cf. Preda and Voigt, 2015). Yet, it is hardly imaginable that a nascent institution—still precarious in its status at the time of drafting its constitution including the health definition—would intend to basically take over the whole established welfare state agenda and indeed even to expand it by making perfect health a political aim. This is even less credible, as one of the global health institutions predating the WHO, the *League of Nations Health Organization*, had come under fire for its alleged political overreach during these times of increasing national isolationism (Cueto *et al*., 2019: 20ff.). There were, accordingly, strong political reasons not to endorse a perfectionist health definition, or at least to keep such ambitions hidden from plain view, especially in 1946, with very fresh memories of the dangers of totalitarianism being abundant.^10^

A more science-oriented reason as to why the WHO is unlikely to have opted for an account of perfect health is that such an ideal is not measurable. After all, it refers to an abstract point of reference. To quantify the health statuses of populations, scientists need metrics and they need to determine thresholds. In other words, they need to plot health along a scale. If health were only a hypothetical point on a limitless scale, it would hardly be a useful metric for scientific purposes. Again, this is not a decisive reason to reject the perfectionist interpretation of the WHO definition. But there are numerous publications by health scientists who use the WHO definition without running into the mentioned problems (Breslow, 1972; Greenfield and Nelson, 1992). So, it seems that many scientists do not assume the perfectionist health interpretation (see also Ware *et al*., 1981: 621).^11^

In contrast, the holistic health interpretation leads to the following point of view: Health is seen as a state of well-being with numerous aspects—physical, mental and social.^12^ Given these dimensions of well-being, health statuses can be assessed in a combined approach, taking the full range of health-related factors into account. Importantly, health is not a fictional point at the end of the scale, but any point along a scale. Some people might have a comparatively bad health status, some might be in good health; all will be positioned along a spectrum. From the health definition itself, nothing follows as to when health is good enough or so bad that state institutions need to interfere. In other words, important political decisions regarding thresholds of sufficient health are not prejudged if we follow a holistic health definition. Such a perspective is much more amenable to the political remit of the WHO, which ended up with fairly limited interventionist power (cf. Packard, 2016: 99ff.; Larsen, 2022: 123ff.).

The overarching focus of the holistic health interpretation is maintenance of health. It is thereby acknowledged that to counter the various threats to health not only medical means are required, but a dynamic level of physical, mental and social assets. This has been an insight of early public health practitioners. For instance, Henry Sigerist, who evidently had a significant indirect influence on the WHO definition via Raymond Gautier’s draft (Larsen, 2022: 119), had already been concerned with the aim of health maintenance.^13^ This provides a dynamic element in the conceptualisation of health, which is also implicit in the WHO definition, despite its reference to a *state*, which seemingly suggests a static view. When Sigerist writes that ‘health is more than the absence of disease’ (Sigerist, 1932: 293), this is meant as a conclusion to an argument acknowledging the environmental and social determinants of health. His point becomes quite clear in a later quote:

A healthy individual is a man [*sic!*] who is well balanced bodily and mentally, and well adjusted to his physical and social environment. He is in full control of his physical and mental faculties, can adapt to environmental changes, so long as they do not exceed normal limits; and contributes to the welfare of society according to his ability. Health is, therefore, not simply the absence of disease: it is something positive, a joyful attitude toward life, and a cheerful acceptance of the responsibilities that life puts upon the individual (Sigerist, 1941: 100).^14^

Sigerist’s terminology, referring to being well balanced, adjusted and in full control, is not aiming towards an ideal of perfection. Rather, he is stating several elements of a good human life within the limits of reality. He believes that health enables an affirmative view of individuals towards their life, not unlimited happiness.

In this section, I have discussed the WHO definition partly from an analytical point of view, in that I distinguished two possible interpretations, a perfectionist and a holistic account of health. I have added historical information regarding the drafting period. Both analytical and historical reasons speak in favour of my thesis that the WHO definition should be read as defining health in a holistic way. Health as complete well-being refers to the full range of factors determining a specific disposition of people to prevent ill health (cf. Ware *et al*., 1981). This ties in nicely with a more recent official statement by the WHO, the Ottawa Charter, which I will cite as final support of my thesis: ‘[H]ealth is a resource for everyday life, not the objective of living. Health is a positive concept emphasizing social and personal resources, as well as physical capacities’ (WHO, 1986). Health is not the best possible state of well-being but a multifarious instrument, including external as well as internal resources, to pursue a good life.

## Why We Need to Distinguish Between Holistic Health and Perfect Health

I have not argued that a conceptualisation of perfect health is wrong-headed or even harmful. Rather, I claimed that perfect health is not the notion that the WHO has been after. It is of import to distinguish between the two notions of health introduced earlier, because confusing them will lead to cross-purposes, not merely in respect to the WHO definition. In this section I will take a closer look at the two health conceptions and discuss the purposes which they can serve. I will also hint at problems with both interpretations that might eventually call for terminological reform.

Holistic health allows to pursue multiple political and economic purposes. For instance, it enables comparisons between groups of people and is especially adept to highlight social inequalities that have an impact on population health. This makes it more pertinent for political purposes than a negative conceptualisation of health as the absence of disease. The latter is absolute or non-comparative and hence does not allow for any interesting information about health-related inequalities between persons.

Importantly, in contrast to perfect health, the scope of holistic health can be contoured by thresholds. As explained, complete well-being can be understood as having all elements that are constitutive of it. What exactly that means in relation to health is of course contested, and I have already insinuated that the WHO did not set a threshold, perhaps intentionally. Still, the required level of holistic health could be determined via political decision-making processes. This makes holistic health open for different substantial interpretations and hence political ambitions.

Despite these advantages, the conceptualisation of health as holistic health has serious drawbacks.^15^ Most significantly, the distinction between health conditions and determinants of health becomes blurry (Bickenbach, 2017: 968, 968; van Druten *et al*., 2022: 2).^16^ Environmental and social determinants of health come with certain probabilities, sometimes unknown, to fall ill or to stay healthy, but they are not constituents of medical conditions themselves; rather, they are their presumed causes (Whitbeck, 1981: 617). As we have seen in the previous example of miners’ health, a poor health disposition is not the same as being unhealthy, that is, suffering from disease or illness.^17^

The potential confusion between poor health dispositions and disease or illness leads to normative confusion as well, especially when we are assessing claims of justice. Disease has a different normative status than a relatively bad health disposition. Arguably, disease has an immediate urgency in relation to human needs, in terms of threatening or involving harm. A comparatively high propensity to fall ill or membership in a vulnerable population as such does not obviously have such normative urgency. Important normative discussions about health justice are short-circuited if we transfer direct urgency to alleviating relatively poor holistic health statuses without thinking about the impact on the lives of real people and merely consider relative positions.

One way forward would be to acknowledge the basic insights of a holistic conceptualisation of health but to nevertheless distinguish between health as a condition of an individual and health-related traits and circumstances that have an impact on the maintenance of individual and population health. We would accordingly need a more adequate term than *health* for combining both of these aspects—an organismic condition, that is, health in the more narrowly medical sense, and a set of health-related resources. Such a revisionary conceptual perspective can only be alluded to here (see Davies and Schramme, 2022).

Accounts of perfect health have a different purpose than accounts of holistic health. The former set an ideal; an ambitious target for individual or social aspiration. According to this perspective, a person can always be potentially healthier, because there is no fixed point on a scale which suffices for health. It seems to me that such an interpretation of health is fully adequate for specific purposes, for instance, introducing a utopian goal and to stop people from becoming complacent about an important element of a good human life. Perfect health shares features with traditional accounts of the virtues, although it is not itself supposed to be a virtue. Virtues are similar to perfect health in that they describe human excellences. Virtues are excellences of character, or perfect dispositions to act fully adequately; health is excellence in relation to well-being, or a perfect organismic disposition to keep harmful and disadvantageous conditions at bay. Becoming virtuous can be an aspiration for human beings and so can becoming perfectly healthy.

However, there is a danger of imposing such an ideal on everyone. If we always have to strive for more health, then we might lose sight of other values, such as pursuing friendships, taking risks or enjoying unhealthy choices. This is a real risk in many modern societies, where health has been turned into a kind of religion and individual mission (Katz, 1997). Socially, similar developments can be studied in relation to so-called ‘healthism’ and generally the moralisation of health (Conrad, 1992).^18^ The problems intensify if health dispositions and risk factors are not clearly distinguished from health conditions. Every single action a person pursues might have an impact on their health, according to the perfectionist health account. Hence, if combined with a prescriptive reading of the ideal—as something to be sought—then health can turn into a totalitarian imperative. This would clearly undermine the initial purpose of setting an ideal.

Whether perfect health will fail to meet its purposes will be established by experience and through history. It is not a necessary feature of the account. As mentioned, there are warning signs. However, more importantly, there is a need to clearly distinguish between holistic health and perfect health because perfect health, in contrast to holistic health, should never be the remit of state institutions.

## Conclusions

‘Health is a state of complete physical, mental and social well-being and not merely the absence of disease or infirmity’ (WHO, 1948: 100). This definition allows for two different interpretations. A perfectionist account, where health describes a hypothetical, perfect state of well-being, or a holistic account, where health is a state of exhaustive well-being, including all relevant dimensions of its constitutive elements. I have argued that the WHO intended to support a holistic account. I provided analytical and historical reasons for this point of view.

To distinguish between the two interpretations of health is important for systematic reasons as well, not merely in relation to the proper interpretation of the WHO’s definition of health. The two different accounts serve different purposes and run into different types of problems, as I have highlighted in this paper. Still, both are perfectly valid notions of health.

## Footnotes

1 There can, of course, be even more than just these two conceptualisations of health. For instance, many would probably define health simply in terms of the absence of disease or illness. Indeed, one of the reasons why the WHO definition has raised concerns is probably due to its explicit diversion from the widespread conceptualisation in negative terms, that is, as absence of something.

2 The *Oxford Dictionary of English* (2015) entry on the adjective forms of *complete* states: ‘1. having all the necessary or appropriate parts: *a complete list of courses offered by the university* | *no woman’s wardrobe is complete without this pretty top* ( … ) 2. [attributive] (often used for emphasis) to the greatest extent or degree; total: *a complete ban on smoking* | *their marriage came as a complete surprise to me*’.

3 The term *holistic* has been used in relation to health by Lennart Nordenfelt (see Nordenfelt, 1995: 12ff., 35ff.). By using this term, I do not want to claim that Nordenfelt endorses the WHO definition.

4 A slightly different distinction between two meanings of the concept of complete—*complete* in an ‘all-or-nothing sense’ and in a sense that ‘admits of degrees’—has been drawn by Sissela Bok in relation to the WHO definition (Bok, 2008: 592). In passing, I also want to note that the label *perfectionist* is of course not supposed to refer to perfectionism in value theory, where it denotes an objective theory of the good.

5 Possibly the first philosopher of medicine to take note of this feature and the likely consequences was Owsei Temkin: ‘I do not think that I read too much into this formula [the WHO definition] if I believe that it tends to include moral values and to identify health with happiness. ( … ) But is the pursuit of happiness itself wholly a medical matter? Our life has many values and ( … ) happiness can sometimes be achieved at the sacrifice of health. ( … ) [I]f health is defined so broadly as to include morality, then the danger exists that the physician will also be burdened with all the duties of the medieval priest’ (Temkin, 1949: 20).

6 This needs to be qualified, because the term *social* was introduced fairly late in the drafting process. However, the point I am making here is to do with the fact that elements of well-being had been listed for some time during the drafting period and that the word *complete* was added to characterise these elements jointly.

7 The Technical Preparatory Committee itself relied on earlier drafts of senior members of related institutional bodies, especially the League of Nations Health Organization (Larsen, 2022). Larsen gives a detailed account of the origins of the WHO definition, tracing it back to Henry Sigerist’s influential publications in the history, sociology and philosophy of medicine, dating mainly from the 1930ies. Sigerist’s ideas were not revisionary or highly original, though, at least not in its focus on positive health. The idea that health includes elements that cannot be captured by the phrase ‘absence of disease’ goes back to antiquity. Especially the notion of health as a form of equilibrium and—in modern terms—resilience has been known for centuries (Edelstein, 1967: 303ff.). So, even if Sigerist’s work probably had a role in finding the relevant formulations, the underlying ideas had been prevalent.

8 One of the members of the Technical Preparatory Committee, Szeming Sze, recalled 40 years later that James H.S. Gear ‘improved the wording’ (WHO, 1988: 33). However, there is no identifiable evidence to corroborate Sze’s recollection.

9 The notion of well-being here is a state of a person including their circumstances. It should not be interpreted as a mental state only, that is, as a kind of feeling.

10 It should also not be forgotten that the early focus of public health institutions, including the precursors of the WHO, was on the prevention of diseases, specifically communicable diseases. This speaks against assuming a focus on health enhancement.

11 Indeed, numerous researchers claim that although the WHO definition sets a political ambition, its main purpose is to set a framework that makes health measurable (Salomon *et al*., 2003; Rubinelli *et al*., 2018; cf. Chatterji *et al*., 2002).

12 In line with this reading, in more recent years, there was also a discussion in the WHO whether to add spiritual well-being to the definition (WHO, 1997: 2; cf. Larson, 1996; Nordenfelt, 2016: 214). The discussion around a fourth aspect of well-being did not lead to official changes, though.

13 Bok also mentions that Sigerist was a close friend of Štampar’s, who was—as mentioned earlier—a member of the drafting group (Bok, 2008: 594).

14 Georges Canguilhem similarly declared that ‘[h]ealth is a set of securities and assurances ( … ), securities in the present, assurances for the future’ (Canguilhem, 1966: 198).

15 Surely not everyone would see the political negotiability of adequate health thresholds as an advantage. However, I am here concerned with a relative advantage over the perfectionist account of health.

16 Once the determinants of health are confused with health itself, there is an additional danger of conceptualising immorality and incivility as forms of health disruptions (cf. Farley 2008: 56). WHO officials were not immune to this problem. For instance, in a memorandum called *International Health of the Future* (1943), Gautier wrote: ‘For health is more than the absence of illness; the word health implies something positive, namely physical, mental, and moral fitness. This is the goal to be reached’ (Larsen, 2022: 117; see also Chisholm, 1946: 16; cf. Cueto *et al*., 2019: 33).

17 The otherwise philosophically important distinction between disease and illness does not matter for the purposes of my essay. I use the terms interchangeably for ease of reading.

18 An important and still highly recommendable early critique of the utopian standard of health is Rene Dubos’s *Mirage of Health* (Dubos, 1959).
